# Upregulated GABA Inhibitory Function in ADHD Children with Child Behavior Checklist–Dysregulation Profile: 123I-Iomazenil SPECT Study

**DOI:** 10.3389/fpsyt.2015.00084

**Published:** 2015-06-02

**Authors:** Shinichiro Nagamitsu, Yushiro Yamashita, Hitoshi Tanigawa, Hiromi Chiba, Hayato Kaida, Masatoshi Ishibashi, Tatsuyuki Kakuma, Paul E. Croarkin, Toyojiro Matsuishi

**Affiliations:** ^1^Department of Pediatrics and Child Health, Kurume University School of Medicine, Fukuoka, Japan; ^2^Department of Radiology, Kurume University School of Medicine, Fukuoka, Japan; ^3^Department of Psychiatry, Kurume University School of Medicine, Fukuoka, Japan; ^4^Biostatistics Center, Kurume University School of Medicine, Fukuoka, Japan; ^5^Department of Psychiatry and Psychology, Mayo Clinic, Rochester, MN, USA

**Keywords:** CBCL-dysregulation profile, iomazenil, GABA, ADHD

## Abstract

The child behavior checklist–dysregulation profile (CBCL–DP) refers to a pattern of elevated scores on the attention problems, aggression, and anxiety/depression subscales of the child behavior checklist. The aim of the present study was to investigate the potential role of GABA inhibitory neurons in children with attention deficit/hyperactivity disorder (ADHD) and dysregulation assessed with a dimensional measure. Brain single photon emission computed tomography (SPECT) was performed in 35 children with ADHD using 123I-iomazenil, which binds with high affinity to benzodiazepine receptors. Iomazenil binding activities were assessed with respect to the presence or absence of a threshold CBCL–DP (a score ≥210 for the sum of the three subscales: Attention Problems, Aggression, and Anxiety/Depression). We then attempted to identify which CBCL–DP subscale explained the most variance with respect to SPECT data, using “age,” “sex,” and “history of maltreatment” as covariates. Significantly higher iomazenil binding activity was seen in the posterior cingulate cortex (PCC) of ADHD children with a significant CBCL–DP. The Anxiety/Depression subscale on the CBCL had significant effects on higher iomazenil binding activity in the left superior frontal, middle frontal, and temporal regions, as well as in the PCC. The present brain SPECT findings suggest that GABAergic inhibitory neurons may play an important role in the neurobiology of the CBCL–DP, in children with ADHD.

## Introduction

Severe behavioral and affective dysregulation with symptoms, such as hyperactivity, aggression, irritability, mood instability, and anxiety, contribute to significant academic and psychosocial impairment in children. Some of these symptoms are consistent with attention deficit hyperactivity disorder (ADHD). ADHD is the most frequent neuropsychiatric disorder in children and often presents with co-occurring disruptive behavior disorders, anxiety disorders, and bipolar disorder. Hyperactivity, irritability, and impulsivity place children at risk of maltreatment as a result of strained parent–child interactions (1, 2). The insecure parent–child relationship further exacerbates the behavioral and affective dysregulation observed in children.

The child behavior checklist-dysregulation profile (CBCL–DP) refers to a pattern of elevated scores on the Attention Problems, Aggression, and Anxiety/Depression subscales of the child behavior checklist (CBCL) (3). The CBCL–DP was originally proposed as a means of identifying youth with bipolar disorder (4). However, recent studies suggest that the results of the CBCL–DP are not simply an early manifestation of a single disease process, but rather that the CBCL–DP can be used as a developmental risk marker for a persisting deficit in self-regulation of affect and behavior (5, 6). The CBCL–DP may be best interpreted as an indicator of symptom severity and functional impairment (7, 8). Children with ADHD who had a threshold level CBCL–DP score (≥210) showed higher rates of comorbidity disorders, including oppositional defiant disorder (ODD), conduct disorder (CD), anxiety disorder, bipolar disorder, and depression (9). The CBCL–DP is also associated with mood, anxiety, disruptive behavior disorders, and substance use in adulthood (3).

The underlying neurobiological defects or aberrant neuronal activity leading to the dysregulation profile in children with ADHD are elusive. Reducing serotoninergic function in children with ADHD and a significant CBCL–DP resulted in slower cognitive performance compared to children with ADHD who did not have the CBCL–DP, indicating that serotoninergic function could play a decisive role in the etiology of the CBCL–DP (10). In addition, the CBCL subscale of “Aggression” was found to be the main discriminator of ADHD children with CBCL–DP versus those without CBCL–DP with respect to serotoninergic dysfunction. Conversely, prior translational work with magnetic resonance spectroscopy and transcanial magnetic stimulation paradigms suggest that GABAergic neurochemistry and neurotransmission are dysregulated in children with ADHD (11). Ongoing work also suggests that defects in the GABAergic system in adults increase an individual’s vulnerability to severe psychiatric illnesses due to aberrant regulation of serotoninergic and/or dopaminergic neurons (12, 13). Previous biochemical and pharmacological studies indicate that deficits in GABA receptor function, induced by intravenous infusion of iomazenil followed by a serotoninergic agonist, predispose healthy volunteers to increased anxiety and dissociative disturbances, suggesting that deficits in the GABAergic system may contribute to the pathophysiology of serotonin-induced psychosis (12).

123I-iomazenil is a radioactive ligand for central-type benzodiazepine receptors that forms a complex with GABA(A) receptors. Thus, 123I-iomazenil single photon emission computed tomography (SPECT) can indirectly index GABA receptor function. 123I-iomazenil is a frequently used radionuclide tracer for presurgical evaluation of patients with refractory partial epilepsy (14, 15). Moreover, recent neuroimaging studies have explored the role of GABAergic inhibitory function in psychiatric disorders such as schizophrenia, anxiety disorders, and developmental disorders (16–20). To our knowledge, there is no previous work which characterizes GABA receptor functioning with 123I-iomazenil SPECT among children with ADHD. The working hypothesis of the present study was that behavioral and affective symptoms in children with ADHD, reflected in CBCL–DP scores, would correlate with changes in cortical GABAergic neuronal activity. To confirm this hypothesis, brain SPECT was performed using 123I-iomazenil in ADHD children with or without CBCL–DP. Further, we tried to identify which of the three significant scales in the CBCL–DP explains the most variance with respect to SPECT data using “age,” “sex,” and “history of maltreatment” as covariates.

## Materials and Methods

### Ethics statement

The design of the study and procedures for obtaining informed consent were approved by the Medical Ethics Committee of Kurume University School of Medicine (#10081). Informed consent was obtained from each child and his/her parents prior to their participation in the study.

### Participants

Thirty-five children with ADHD (23 boys, 12 girls) enrolled in the study. Participants were recruited after visits from the Department of Pediatrics, Kurume University, for the management of externalizing symptoms (e.g., difficulty maintaining attention, restlessness, hyperactivity, and aggressive behavior) or internalizing symptoms (e.g., anxiety, dissociation, and depressive symptoms). A diagnosis of ADHD was made using the *Diagnostic and Statistical Manual of Mental Disorders, 4th Edition, Text Revision (Dsm-Iv-Tr)* (21). Children who had anxious or depressive symptoms, but did not have ADHD symptoms were excluded in this study. Of the 35 child participants with ADHD, 15 had the combined type, 11 had hyperactive-impulsive type, and 9 had inattentive type. Seventeen children (7 male, 10 females) had experienced an obvious maltreatment, such as physical (*n* = 9), psychological (*n* = 6), or sexual abuse (*n* = 1), or sexual assault (*n* = 1) during preschool. In 11 of these instances, a child-welfare consultation center had previously supported the families in hopes of preventing maltreatment. Two children stayed in a child residential care institution. However, none of the participants met the diagnostic criteria for posttraumatic stress disorder (PTSD) on assessment. Seven of the 35 subjects (20%) had comorbid disorders, such as ODD (*n* = 2), CD (*n* = 1), anxiety disorder (*n* = 2), and depression (*n* = 2). The mean age of the children at the time of their hospital visit was 10.4 years. All participants were medication naïve prior to enrollment.

### Child behavior checklist

Behavioral and psychiatric assessments of the children included the CBCL, ADHD rating scale (hyperactivity/impulsive and inattention scores, as well as total score) (22, 23), the Child Depression Inventory (CDI), the Child Dissociative Checklist (CDC), and the Wechsler Intelligence Scale for Children (WISC-III). The CBCL was used to evaluate children’s emotional and behavioral functioning, competencies, and social problems, with specific items evaluating internalizing and externalizing symptoms, as well as attention and thought problems. Items evaluating internalizing symptoms focus on withdrawal, somatic complaints, and anxiety/depression. Items evaluating externalizing symptoms focus on delinquent or aggressive behavior. The CBCL–DP refers to a pattern of elevated scores on the Attention Problems, Aggression, and Anxiety/Depression subscales of the CBCL. A threshold CBCL–DP was defined as a score ≥210 for the sum of three subscales. (4) Physicians rated the participants using the ADHD rating scale, CDI, and CDC, and the parents rated their children using the CBCL.

### Iomazenil single photon emission computed tomography and analysis of regions of interest

**Figure 1:**
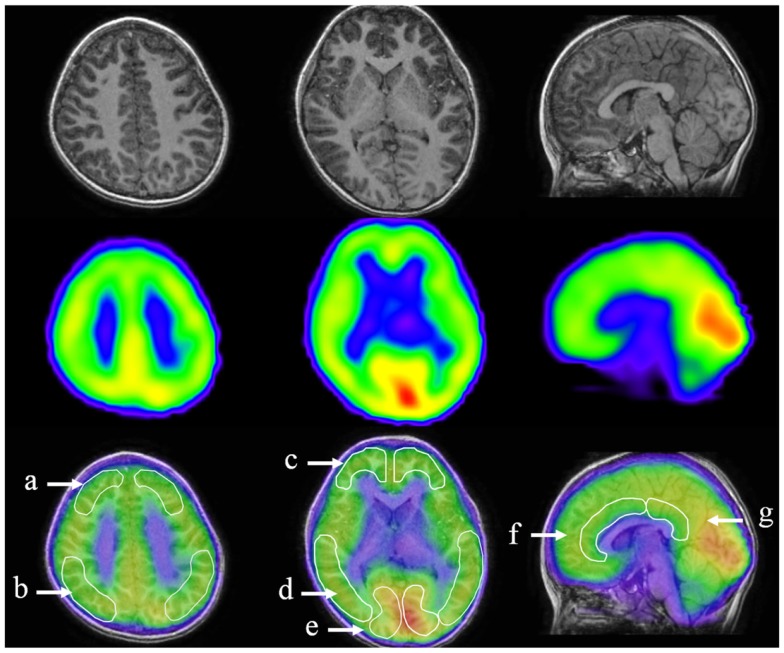
**Designated regions of interest (ROIs) in fusion images of 123I-iomazenil SPECT and MRI**. The top panel shows brain MRI (transverse and sagittal T_1_ sequences), the middle panel shows corresponding results of 123I-iomazenil SPECT, and the bottom panel shows fusion imaging. Outlined regions in the bottom panel indicate designated ROIs, namely (a) the superior frontal, (b) parietal, (c) middle frontal, (d) temporal, (e) occipital regions, (f) anterior, and (g) posterior cingulate gyrus.

All 35 children underwent iomazenil SPECT imaging of the brain. Briefly, children were injected intravenously with a bolus of 95–117 MBq 123I-iomazenil (Nihon Medi-Physics, Tokyo, Japan), which binds with high affinity to benzodiazepine receptors. The SPECT scan was performed 3 h after injection of the tracer, without any sedation, using a large field-of-view dual-detector camera and a computer system equipped with a low-energy, high-resolution, parallel-hole collimator. The dual detector camera rotated over 180° in a circular orbit and in 32 steps of 40 s each to cover 360° in about 22 min. Brain magnetic resonance imaging (MRI) was performed using a superconducting magnet operating at 1.5 T. For coregistered SPECT and MRI analysis, a method of image integration was applied using Fusion Viewer software (Nihon Medi-Physics) with a registration algorithm based on maximum mutual information (Figure 1). Subsequently, the cortical and subcortical regions of interest (ROIs) in the acquired SPECT data were defined. Using elliptical templates, the ROIs were placed over the following regions: the superior frontal, middle frontal, parietal, temporal, and occipital regions in each hemisphere; the midbrain; and the anterior and posterior cingulate cortex (ACC and PCC, respectively; Figure 1). Each relative iomazenil binding activity in ROIs was expressed as a ratio of that in the occipital cortex. As 123I-iomazenil affinity in the occipital region was maximum and stable in brain cortex, the occipital region was used as a reference (24).

### Data analysis

The differences of each CBCL subscale, ADHD-RS, CDI score, CDC score, and Intelligence scale between ADHD children with/without CBCL–DP were compared by student’s *t*-test. We first analyzed correlations between the relative iomazenil binding activity expressed as a ratio in each ROI and psychometric profiles after controlling for the effects of age, sex, and history of maltreatment. Further, we compared iomazenil binding activity with respect to the presence or absence of a threshold CBCL–DP score in these children and tried to identify which of the three CBCL–DP subscales explained the most variance with respect to the SPECT data. Associations between each of the CBCL–DP subscales and iomazenil binding activity in each brain area were evaluated using liner regression models, with “age,” “sex,” and “history of maltreatment” as covariates.

## Results

Behavioral and psychiatric assessments of the participants were shown in Table 1. Of the 35 participants, 15 had a threshold CBCL–DP score (i.e., a score ≥210) and 20 had CBCL–DP scores <210. The group with threshold CBCL–DP scores had a lower ratio of male to female participants and more instances of maltreatment. Each ADHD rating scale, all subscales of CBCL with the exception of somatic problems, and CDC score were significantly higher in ADHD children with threshold CBCL–DP scores. Four participants with threshold level CBCL–DP scores had comorbidity disorders, including depression (*n* = 2), ODD (*n* = 1), and CD (*n* = 1). Three participants without threshold CBCL–DP scores had comorbidity disorders, including anxiety disorder (*n* = 2) and ODD (*n* = 1). There was a difference in the CBCL–DP scores between participants with/without comorbidity disorders; however, this difference did not reach statistical significance (*n* = 28, 199 ± 20, and *n* = 7, 209 ± 12, respectively, *p* = 0.10).

**Table 1:** **Behavioral and psychiatric assessments of participants**.

	ADHD children without significant CBCL–DP	ADHD children with significant CBCL–DP
Number of participants	20	15
Male:Female	17:3	6 ± 9
Mean age (years)	10.7 ± 1.9	10.0 ± 1.8
Experience of maltreatment (%)	40	80
ADHD-RS		
Total score	23.5 ± 9.2	30.6 ± 9.0*
Inattention score	14.8 ± 5.7	17.9 ± 4.9*
Impulsivity/hyperactivity score	9.2 ± 5.6	12.6 ± 6.0*
CBCL score		
Internalizing score	64.4 ± 9.7	80.8 ± 8.2**
Externalizing score	62.7 ± 8.5	71.1 ± 4.8**
Aggressive behaviors	63.7 ± 9.0	79.2 ± 7.2**
Anxious/depressed	58.1 ± 4.9	66.4 ± 4.5**
Attention problem	66.2 ± 5.4	73.7 ± 5.5**
Delinquent behavior	62.7 ± 8.5	71.1 ± 4.8**
Withdrawn	61.5 ± 5.6	66.3 ± 5.6**
Somatic problems	55.5 ± 15.2	58.4 ± 9.5
Social problems	62.0 ± 8.8	66.9 ± 8.1*
Thought problems	59.9 ± 9.7	67.8 ± 9.9*
CDI score	14.3 ± 9.6	15.6 ± 6.3
CDC score	6.7 ± 3.8	13.3 ± 4.7**
WISC-III	90.0 ± 15.6	87.3 ± 13.7

*CBCL–DP, child behavior checklist–dysregulation profile; ADHD-RS, attention deficit hyperactivity disorder rating scale; CDI, child depression inventor; CDC, children dissociative checklist; WISC, Wechsler intelligence scale for children*.

Significant difference from children without significant CBCL–DP (*indicates *p* < 0.05, **indicates *p* < 0.001).

**Figure 2:**
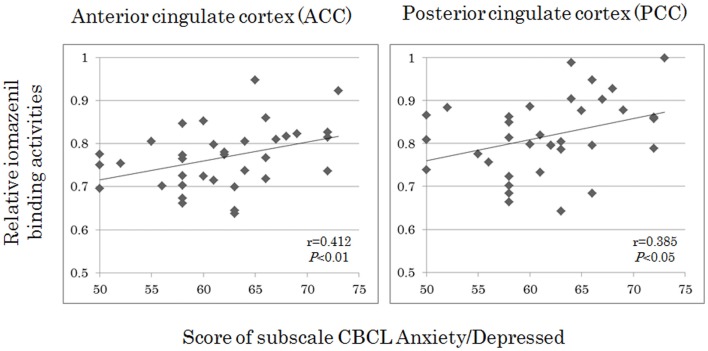
**Correlations between iomazenil binding activity in the anterior and posterior cingulate cortices and score of subscale CBCL anxiety/depressed in all subjects**. The higher relative iomazenil binding activities in ACC and PCC are significantly associated with the higher scores of “Anxiety/Depressed” subscale in the CBCL. CBCL, child behavior checklist.

Analyses of all participants (*n* = 35) revealed correlations between iomazenil binding activity in several brain regions and some part of the CBCL profile, after controlling for the effects of age, sex, and a history of maltreatment (Table 2). In both ACC and PCC, iomazenil binding activity had a statistically significant positive correlation with scores on the Anxiety/Depressed (Table 2; Figure 2), Internalizing, and Withdrawal Problems subscales of the CBCL (Table 2). In addition, significant positive correlations were noted for iomazenil binding activity in the ACC and Thought Problems on the CBCL, as well as for iomazenil binding activity in the PCC and Attention problems and Social Problems on the CBCL (Table 2). These significant correlations were not seen for other combinations in other brain regions, except for iomazenil binding activity in the midbrain and Thought Problems on the CBCL, and iomazenil binding activity in the right temporal region and Social Problems on the CBCL. There were no significant correlations between iomazenil binding activities in any brain region and any of the ADHD rating scales, CDI score, and CDC score (data not shown). Iomazenil binding activity in the PCC was significantly higher in ADHD children with a threshold CBCL–DP score than in ADHD children with scores <210 after controlling for the effects of age, sex, and a history of maltreatment (Table 3, *F*-value = 4.36, *p* < 0.05). Of the three CBCL–DP subscales, the Anxiety/Depression subscale had significant effects on higher iomazenil binding activity in the left superior frontal, middle frontal, and temporal regions, as well as in the PCC (Table 4).

**Table 2:** **Partial correlation coefficients between CBCL profiles and the iomazenil binding activites in each brain region**.

	Superior frontal		Parietal		Middle frontal		Temporal		Mid brain	ACC	PCC
	R	L	R	L	R	L	R	L			
CBCL profiles
Total problems	0.341	0.237	0.192	0.267	0.301	0.229	0.284	0.191	0.178	0.385	0.245
Internalizing problems	0.199	0.160	0.026	0.068	0.110	0.188	0.136	0.332	0.274	0.477*	0.536**
Externalizing problems	0.158	0.086	0.057	0.090	0.148	0.027	0.127	−0.072	0.032	0.078	−0.041
Aggressive behaviors^a^	0.119	0.052	0.036	0.077	0.113	−0.042	0.088	−0.111	−0.040	0.017	−0.026
Anxious/depressed^a^	0.309	0.284	0.120	0.129	0.264	0.335	0.246	0.369	0.355	0.536**	0.524**
Attention problem^a^	0.265	0.120	0.234	0.157	0.228	0.166	0.248	0.160	0.212	0.378	0.483**
Delinquent behavior	0.122	0.091	0.068	0.102	0.133	0.087	0.175	0.108	0.159	0.077	0.008
Withdrawn	0.278	0.105	0.163	0.006	0.192	0.057	0.338	0.214	−0.012	0.425*	0.509**
Somatic problems	−0.131	0.045	−0.162	0.035	−0.133	0.074	−0.246	0.080	0.216	−0.050	0.205
Social problems	0.304	0.112	0.325	0.217	0.264	0.108	0.406*	0.204	0.129	0.383	0.440*
Thought problems	0.322	0.221	0.269	0.207	0.307	0.320	0.310	0.239	0.546**	0.432*	0.360

*CBCL, child behavior checklist; ACC, anterior cingulate cortex; PCC, posterior cingulate cortex; R, right; L, left*.

*Indicates *p* < 0.05; **indicates *p* < 0.01.

*^a^Indicates subscale which comprises CBCL–DP (dysregulation profile)*.

**Table 3:** **Effect of the significant CBCL–DP (score ≥210) on iomazenil binding activities in each brain region**.

Brain area	Effect	*F*-Test	*p*-Value
Right superior frontal	Significant CBCL–DP	0.88	0.35
Left superior frontal	Significant CBCL–DP	0.18	0.67
Right parietal	Significant CBCL–DP	2.07	0.16
Left parietal	Significant CBCL–DP	0.37	0.55
Right middle frontal	Significant CBCL–DP	1.57	0.22
Left middle frontal	Significant CBCL–DP	0.44	0.51
Right temporal	Significant CBCL–DP	1.26	0.27
Left temporal	Significant CBCL–DP	0.05	0.82
Midbrain	Significant CBCL–DP	0.2	0.66
Anterior cingulate cortex	Significant CBCL–DP	0.99	0.33
Posterior cingulate cortex	Significant CBCL–DP	4.36	<0.05

*CBCL–DP, child behavior checklist–dysregulation profile*.

**Table 4:** **Effect of the CBCL–DP subscale on iomazenil binding activities in each brain region**.

Brain area	Effect	*F*-Test	*p*-Value
Right superior frontal	Aggressive behavior	0.03	0.8708
	Anxious/depressed	2.79	0.1058
	Attention problem	0.12	0.7268
Left superior frontal	Aggressive behavior	0.01	0.9374
	Anxious/depressed	4.28	0.0479*
	Attention problem	1.6	0.2157
Right parietal	Aggressive behavior	0	0.9675
	Anxious/depressed	0.13	0.7249
	Attention problem	0.39	0.5367
Left parietal	Aggressive behavior	0.33	0.5703
	Anxious/depressed	0.67	0.4214
	Attention problem	0.02	0.8843
Right middle frontal	Aggressive behavior	0	0.9771
	Anxious/depressed	1.83	0.1871
	Attention problem	0.06	0.8060
Left middle frontal	Aggressive behavior	0.5	0.4873
	Anxious/depressed	5.1	0.0319*
	Attention problem	0.92	0.3457
Right temporal	Aggressive behavior	0.03	0.8709
	Anxious/depressed	1.46	0.2363
	Attention problem	0.02	0.8888
Left temporal	Aggressive behavior	0.12	0.7354
	Anxious/depressed	7.55	0.0104*
	Attention problem	1.91	0.1777
Mid brain	Aggressive behavior	0.66	0.4231
	Anxious/depressed	1.98	0.1699
	Attention problem	0.21	0.6482
Anterior cingulate cortex	Aggressive behavior	0.97	0.3331
	Anxious/depressed	3.94	0.0570
	Attention problem	1.06	0.3118
Posterior cingulate cortex	Aggressive behavior	0.99	0.3277
	Anxious/depressed	5.62	0.0248*
	Attention problem	1.26	0.2714

*CBCL–DP, child behavior checklist–dysregulation profile*.

*Indicates significant effects on higher iomazenil binding activity.

## Discussion

This is the first neuroimaging study showing that behavioral and affective symptoms in children with ADHD, reflected in CBCL–DP scores, are correlated with changes in cortical GABAergic neuronal activity. Overall, increased iomazenil activity in the ACC and PCC was associated with higher scores on many of the CBCL subscales. In ADHD children with a significant CBCL–DP, iomazenil activity was upregulated in the PCC. Of the three CBCL–DP subscales, the Anxiety/Depression subscale had a significant effect on iomazenil binding activity in many brain regions. These results suggest that behavioral and affective dysregulation in ADHD children may be characterized by changes of GABAergic neural activity. In this section, we discuss the role of the cingulate cortex in GABA function, the association between CBCL–DP scores and GABA function, and age-dependent differences in GABA function.

The cingulate cortex is one of the largest parts of the limbic lobe and the prefronto-limbic circuitry. The ACC plays key roles in emotion, motivation, and motor functions, whereas the PCC is involved in emotion, facial recognition, and memory functions (25–27). In the present study, we found higher iomazenil binding activity in ACC and PCC that was associated with higher scores on many of the CBCL subscales in ADHD children with and without CBCL–DP. Similar findings have been reported in healthy adults. For example, Kim et al. (28) found a positive correlation in healthy subjects between high GABA concentrations in the ACC and a high harm avoidance temperament, characterized by worrying about potential problems, fearful of uncertainties, and being shy in unfamiliar environments. Moreover, increased activity in the PCC has been observed in emotional disorders, including obsessive–compulsive disorder, major depression, and social phobia (29, 30). Because the cingulate cortex has been suggested to have an important role in modulating human fear and anxiety by modulating the activity of other limbic structures, including the amygdala (31), the increased GABAergic function in the cingulate cortex of ADHD children in the present study may have inhibited excessive excitation of the limbic system, which contributes to the development of behavioral and affective dysregulation.

We found that the Anxiety/Depression subscale of the CBCL–DP explains the most variance with respect to SPECT data in various brain regions using “age,” “sex,” and “history of maltreatment” as covariates. The Aggression and Attention Problem subscales of the CBCL–DP had no significant effects on SPECT data in various brain regions. These findings strongly support previous converging lines of evidence regarding the association between GABAergic activation and increased anxiety (32). Conversely, several biochemical and genetic studies have provided evidence of a significant role of serotoninergic function in aggressive behavior. For example, an inverse correlation has been reported between downregulated platelet or CSF 5-hydroxyindolecetic acid (5-HIAA), a major metabolite of serotonin, and levels of aggression and impulsivity (33, 34). Furthermore, Haberstick et al. (35) reported an association between certain promoter polymorphisms in the serotonin transporter (5HTTLPR) and greater aggressive behavior in middle childhood, suggesting that differences in serotonergic functioning may be a contributing factor to different levels of aggressive behavior. In terms of the biological mechanism underlying attention function, an important role for dopaminergic neurons has been proposed. Several neuroimaging studies have shown aberrant dopamine transporter (DAT) levels in the nucleus accumbens, caudate, and midbrain, as well as a positive relationship between DAT levels in the putamen and inattention scores in ADHD patients (36–38). Together, these findings suggest that changes in several neurotransmitter systems, including serotoninergic, dopaminergic, and GABAergic neurons, are likely to be involved in constructing the clinical manifestations of the CBCL–DP.

Significant positive correlations between GABAergic inhibitory function and the Anxiety/Depression subscale were also seen in our study. Although previous neuroimaging studies have reported those correlations in adulthood with psychiatric disorders (17, 28), the present study is the first report of the correlation in childhood with psychiatric disorders. Despite the positive correlation in childhood, previous neuroimaging studies using iomazenil SPECT have revealed negative correlations between GABA–benzodiazepine receptor binding activity and the severity of anxiety symptoms in adults with panic or traumatic disorders (17–19). It is well known that there are considerable changes in the number of GABA receptors and in subunit expression during brain development (39). Specifically, the greatest number of GABA receptors is found in the youngest children, with numbers decreasing exponentially with age, and there are age-related increases in α1-subunit-containing GABA receptors (40). These age-related changes in GABA receptors may affect outcomes when assessing increases and/or decreases in overall iomazenil binding activity in children.

The present study has several limitations that require consideration in future studies. For example, in the present study, SPECT exhibited poor resolution around some limbic regions, such as the amygdala and hippocampus, which are important for emotion processing. In these small regions, the obtained radioactivity might differ from the true activity because of partial volume effect (PVE). The PVE can be defined as the underestimation of binding per unit brain volume in small objects or regions because of the blurring of the radioactivity (spill-out and spill-in) between regions. These regions need to be resolved using MR imaging-based correction for PVE (41, 42). Brain imaging data from normal healthy children are not available because of ethical concerns with SPECT studies of this population. Therefore, we focused our research questions on the correlation between GABAergic inhibitory function in specific brain regions and psychometric profiles. It is possible that, in addition to the population of people with ADHD, our result is generalizable to the normal population. Furthermore, we selected iomazenil activity in the occipital regions as a reference, meaning that we could not evaluate inhibitory function in occipital regions. We did not clarify how putative dopaminergic or serotoninergic changes are involved in other subscales, such as the Aggression and Attention Problem subscales, of the CBCL–DP. It is possible that investigations incorporating the simultaneous assessment of benzodiazepine receptor binding activity and homovanillic acid (HVA) and 5HIAA in the urine (principal metabolites of dopamine and serotonin, respectively) could provide new insights into the underlying neurobiological defects or aberrant neuronal activity leading to the dysregulation profile in children.

In conclusion, the present 123I-iomazenil brain SPECT study provides evidence that changes in GABAergic inhibitory neuronal activity correlate with some elements of function measured by the CBCL–DP. Brain SPECT may be useful for the evaluation of the possible pathogenesis of neuropsychiatric symptoms observed in children.

## Author Contributions

SN participated in the design of this study and compiled the manuscript. SN, YY, and HC saw the patients and obtained informed consent and their agreement to participate in the study. Diagnosis of comorbidity disorders was made by HC. SN and HC summarized participant behavioral and psychiatric assessments, including CBCL–DP data. Three radiologists (HT, HK, and MI) were in charge of radioactive measurements and calculations of iomazenil activity using ROIs. TK, a statistician, conducted the statistical analyses. PC and TM supervised the preparation of the manuscript.

## Conflict of Interest Statement

The authors declare that the research was conducted in the absence of any commercial or financial relationships that could be construed as a potential conflict of interest.

## References

[1] Weinstein D Staffelbach D Biaggio M Attention-deficit hyperactivity disorder and posttraumatic stress disorder: differential diagnosis in childhood sexual abuse. Clin Psychol Rev (2000) 20:359–78. 10.1016/S0272-7358(98)00107-X 10779899

[2] Ouyang L Fang X Mercy J Perou R Grosse SD. Attention-deficit/hyperactivity disorder symptoms and child maltreatment: a population-based study. J Pediatr (2008) 153:851–6. 10.1016/j.jpeds.2008.06.002 18619612

[3] Althoff RR Verhulst FC Rettew DC Hudziak JJ van der Ende J. Adult outcomes of childhood dysregulation: a 14-year follow-up study. J Am Acad Child Adolesc Psychiatry (2010) 49:1105–16. 10.1016/j.jaac.2010.08.006 20970698 PMC2965164

[4] Biederman J Petty CR Monuteaux MC Evans M Parcell T Faraone SV The child behavior checklist-pediatric bipolar disorder profile predicts a subsequent diagnosis of bipolar disorder and associated impairments in ADHD youth growing up: a longitudinal analysis. J Clin Psychiatry (2009) 70:732–40. 10.4088/JCP.08m04821 19389330 PMC3066229

[5] Holtmann M Buchmann AF Esser G Schmidt MH Banaschewski T Laucht M. The child behavior checklist-dysregulation profile predicts substance use, suicidality, and functional impairment: a longitudinal analysis. J Child Psychol Psychiatry (2011) 52:139–47. 10.1111/j.1469-7610.2010.02309.x 20854363

[6] Meyer SE Carlson GA Youngstrom E Ronsaville DS Martinez PE Gold PW Long-term outcomes of youth who manifested the CBCL-pediatric bipolar disorder phenotype during childhood and/or adolescence. J Affect Disord (2009) 113:227–35. 10.1016/j.jad.2008.05.024 18632161

[7] Peyre H Speranza M Cortese S Wohl M Purper-Ouakil D Do ADHD children with and without child behavior checklist-dysregulation profile have different clinical characteristics, cognitive features, and treatment outcomes? J Atten Disord (2015) 19:63–71. 10.1177/1087054712452135 22837549

[8] McGough JJ McCracken JT Cho AL Castelo E Sturm A Cowen J A potential electroencephalography and cognitive biosignature for the child behavior checklist-dysregulation profile. J Am Acad Child Adolesc Psychiatry (2013) 52:1173–82. 10.1016/j.jaac.2013.08.002 24157391 PMC3839814

[9] Biederman J Petty CR Day H Goldin RL Spencer T Faraone SV Severity of the aggression/anxiety-depression/attention child behavior checklist profile discriminates between different levels of deficits in emotional regulation in youth with attention-deficit hyperactivity disorder. J Dev Behav Pediatr (2012) 33:236–43. 10.1097/DBP.0b013e3182475267 22278125 PMC3319866

[10] Zepf FD Wöckel L Poustka F Holtmann M. Diminished 5-HT functioning in CBCL pediatric bipolar disorder-profiled ADHD patients versus normal ADHD: susceptibility to rapid tryptophan depletion influences reaction time performance. Hum Psychopharmacol (2008) 23:291–9. 10.1002/hup.934 18421802

[11] Edden RA Crocetti D Zhu H Gilbert DL Mostofsky SH. Reduced GABA concentration in attention-deficit/hyperactivity disorder. Arch Gen Psychiatry (2012) 69:750–3. 10.1001/archgenpsychiatry.2011.2280 22752239 PMC3970207

[12] D’Souza DC Gil RB Zuzarte E MacDougall LM Donahue L Ebersole JS Gamma-aminobutyric acid-serotonin interactions in healthy men: implications for network models of psychosis and dissociation. Biol Psychiatry (2006) 59:128–37. 10.1016/j.biopsych.2005.06.020 16140281

[13] Scheel-Krüger J Dopamine-GABA interactions: evidence that GABA transmits, modulates and mediates dopaminergic functions in the basal ganglia and the limbic system. Acta Neurol Scand Suppl (1986) 107:1–54. 3014800

[14] Higurashi N Hamano S Oritsu T Minamitani M Sasaki M Ida H. Iomazenil hyperfixation in single photon emission computed tomography study of malformations of cortical development during infancy. Eur J Paediatr Neurol (2011) 15:372–5. 10.1016/j.ejpn.2011.03.007 21501962

[15] Kuroda H Ogasawara K Aso K Beppu T Kobayashi M Chida K Spontaneous recovery of reduced cortical central benzodiazepine receptor binding potential on I-123 Iomazenil SPECT in a patient with status epilepticus. Clin Nucl Med (2010) 35:126–7. 10.1097/RLU.0b013e3181c7c168 20090466

[16] Verhoeff NP Soares JC D’Souza CD Gil R Degen K Abi-Dargham A [^123^I] Iomazenil SPECT benzodiazepine receptor imaging in schizophrenia. Psychiatry Res (1999) 91:163–73. 10.1016/S0925-4927(99)00027-X 10641580

[17] Geuze E van Berckel BN Lammertsma AA Boellaard R de Kloet CS Vermetten E Reduced GABAA benzodiazepine receptor binding in veterans with post-traumatic stress disorder. Mol Psychiatry (2008) 13:74–8. 10.1038/sj.mp.4002054 17667960

[18] Hasler G Nugent AC Carlson PJ Carson RE Geraci M Drevets WC Altered cerebral gamma-aminobutyric acid type A-benzodiazepine receptor binding in panic disorder determined by [^11^C]flumazenil positron emission tomography. Arch Gen Psychiatry (2008) 65:1166–75. 10.1001/archpsyc.65.10.1166 18838633

[19] Bremner JD Innis RB Southwick SM Staib L Zoghbi S Charney DS. Decreased benzodiazepine receptor binding in prefrontal cortex in combat-related posttraumatic stress disorder. Am J Psychiatry (2000) 157:1120–6. 10.1176/appi.ajp.157.7.1120 10873921

[20] Mori T Mori K Fujii E Toda Y Miyazaki M Harada M Evaluation of the GABAergic nervous system in autistic brain: (123)I-iomazenil SPECT study. Brain Dev (2012) 34:648–54. 10.1016/j.braindev.2011.10.007 22099869

[21] American Psychiatric Association. Diagnostic and Statistical Manual of Mental Disorders. 4th ed. Text Revision (DSM-IV-TR). Washington: American Psychiatric Association (2000).

[22] Achenbach TM Manual for the Child Behavior Checklist/4–18 and 1991 Child Profile. Burlington: University of Vermont Department of Psychiatry (1991).

[23] DuPaul GJ Anastopoulos AD Power TJ Reid R Ikeda MJ McGoey K Parent ratings of attention-deficit/hyperactivity disorder symptoms: factor structure and normative data. J Psychopathol Behav Assess (1998) 20:83–102. 10.1023/A:1023087410712

[24] Laruelle M Abi-Dargham A Rattner Z Al-Tikriti MS Zea-Ponce Y Zoghbi SS Single photon emission tomography measurement of benzodiazepine receptor number and affinity in primate brain: a constant infusion paradigm with [123I]iomazenil. Eur J Pharmacol (1993) 230:119–23. 10.1016/0014-2999(93)90421-D 8381354

[25] Maddock RJ Garrett AS Buonocore MH. Posterior cingulate cortex activation by emotional words: fMRI evidence from a valence decision task. Hum Brain Mapp (2003) 18:30–41. 10.1002/hbm.10075 12454910 PMC6871991

[26] Mega MS Cummings JL Frontal subcortical circuits. In: Salloway SP Malloy PF Duffy JD, editors. The Frontal Lobes and Neuropsychiatrie Illness. Washington: American Psychiatric Publishing (2001). p. 15–32.

[27] Devinsky O Morrell MJ Vogt BA. Contributions of anterior cingulate cortex to behaviour. Brain (1995) 118:279–306. 10.1093/brain/118.1.279 7895011

[28] Kim HJ Kim JE Cho G Song IC Bae S Hong SJ Associations between anterior cingulate cortex glutamate and gamma-aminobutyric acid concentrations and the harm avoidance temperament. Neurosci Lett (2009) 464:103–7. 10.1016/j.neulet.2009.07.087 19660524

[29] Bench CJ Friston KJ Brown RG Scott LC Frackowiak RS Dolan RJ. The anatomy of melancholia – focal abnormalities of cerebral blood flow in major depression. Psychol Med (1992) 22:607–15. 10.1017/S003329170003806X 1410086

[30] McGuire PK Bench CJ Frith CD Marks IM Frackowiak RS Dolan RJ. Functional anatomy of obsessive-compulsive phenomena. Br J Psychiatry (1994) 164:459–68. 10.1192/bjp.164.4.459 8038933

[31] Hahn A Stein P Windischberger C Weissenbacher A Spindelegger C Moser E Reduced resting-state functional connectivity between amygdala and orbitofrontal cortex in social anxiety disorder. Neuroimage (2011) 56:881–9. 10.1016/j.neuroimage.2011.02.064 21356318

[32] Shen Q Fuchs T Sahir N Luscher B. GABAergic control of critical developmental periods for anxiety- and depression-related behavior in mice. PLoS One (2012) 7:e47441. 10.1371/journal.pone.0047441 23071808 PMC3469546

[33] Kruesi MJ Hibbs ED Zahn TP Keysor CS Hamburger SD Bartko JJ A 2-year prospective follow-up study of children and adolescents with disruptive behavior disorders. Prediction by cerebrospinal fluid 5-hydroxyindoleacetic acid, homovanillic acid, and autonomic measures? Arch Gen Psychiatry (1992) 49:429–35. 10.1001/archpsyc.1992.01820060009001 1376104

[34] Golubchik P Mozes T Vered Y Weizman A. Platelet poor plasma serotonin level in delinquent adolescents diagnosed with conduct disorder. Prog Neuropsychopharmacol Biol Psychiatry (2009) 33:1223–5. 10.1016/j.pnpbp.2009.07.003 19596039

[35] Haberstick BC Smolen A Hewitt JK. Family-based association test of the 5HTTLPR and aggressive behavior in a general population sample of children. Biol Psychiatry (2006) 59:836–43. 10.1016/j.biopsych.2005.10.008 16412987

[36] Jucaite A Fernell E Halldin C Forssberg H Farde L. Reduced midbrain dopamine transporter binding in male adolescents with attention-deficit/hyperactivity disorder: association between striatal dopamine markers and motor hyperactivity. Biol Psychiatry (2005) 57:229–38. 10.1016/j.biopsych.2004.11.009 15691523

[37] Spencer TJ Biederman J Madras BK Dougherty DD Bonab AA Livni E Further evidence of dopamine transporter dysregulation in ADHD: a controlled PET imaging study using altropane. Biol Psychiatry (2007) 62:1059–61. 10.1016/j.biopsych.2006.12.008 17511972 PMC2715944

[38] da Silva N Jr Szobot CM Anselmi CE Jackowski AP Chi SM Hoexter MQ Attention deficit/hyperactivity disorder: is there a correlation between dopamine transporter density and cerebral blood flow? Clin Nucl Med (2011) 36:656–60. 10.1097/RLU.0b013e318219b49d 21716015

[39] Rissman RA De Blas AL Armstrong DM. GABA(A) receptors in aging and Alzheimer’s disease. J Neurochem (2007) 103:1285–92. 10.1111/j.1471-4159.2007.04832.x 17714455

[40] Chugani DC Muzik O Juhász C Janisse JJ Ager J Chugani HT. Postnatal maturation of human GABAA receptors measured with positron emission tomography. Ann Neurol (2001) 49:618–26. 10.1002/ana.1003 11357952

[41] Kato H Matsuda K Baba K Shimosegawa E Isohashi K Imaizumi M MR imaging-based correction for partial volume effect improves detectability of intractable epileptogenic foci on iodine 123 iomazenil brain SPECT images: an extended study with a larger sample size. AJNR Am J Neuroradiol (2012) 33:2088–94. 10.3174/ajnr.A3121 22627794 PMC7965582

[42] Kato H Shimosegawa E Oku N Kitagawa K Kishima H Saitoh Y MRI-based correction for partial-volume effect improves detectability of intractable epileptogenic foci on 123I-iomazenil brain SPECT images. J Nucl Med (2008) 49:383–9. 10.2967/jnumed.107.046136 18287271

